# Evaluating the Impact of Breastfeeding on Rotavirus Antigenemia and Disease Severity in Indian Children

**DOI:** 10.1371/journal.pone.0146243

**Published:** 2016-02-01

**Authors:** Sushmita Das, Ganesh Chandra Sahoo, Pradeep Das, Utpal Kant Singh, Anil Kumar Jaiswal, Prachi Singh, Ranjeet Kumar, Rishikesh Kumar

**Affiliations:** 1 Department of Microbiology, All-India Institute of Medical Sciences (AIIMS), Patna, India; 2 Virology Unit, Rajendra Memorial Research Institute of Medical Sciences (ICMR), Patna, India; 3 Department of Paediatrics, Nalanda Medical College and Hospital, Patna, India; University of North Carolina School of Medicine, UNITED STATES

## Abstract

**Objectives:**

To evaluate the contribution of breastfeeding to Rotavirus (RV)-induced antigenemia and/or RNAemia and disease severity in Indian children (<2 yrs age).

**Methods:**

Paired stool and serum samples were collected from (a) hospitalized infants with diarrhea (n = 145) and (b) healthy control infants without diarrhea (n = 28). Stool RV-antigen was screened in both groups by commercial rapid-test and enzyme immunoassay. The disease severity was scored and real-time-PCR was used for viral-load estimation. Serum was evaluated for RV-antigenemia by EIA and RV-RNAemia by RT-PCR. Data was stratified by age-group and breastfeeding status and compared.

**Results:**

Presence of RV-antigenemia and RV-RNAemia was positively related with presence of RV in stool. Disease severity and stool viral-load was significantly associated with RV-antigenemia[(r = 0.74; CI:0.66 to 0.84; P<0.0001,R^2^ = 0.59) and (r = -0.55; CI:-0.68 to -0.39; P<0.0001,R^2^ = 0.31) respectively], but not with RV-RNAemia. There was significant reduction in RV-antigenemiarate in the breast-fed group compared to non-breastfed infants, especially in 0–6 month age group (P<0.001). Non-breastfed infants were at risk for RV-antigenemia with severe disease manifestations in form of high Vesikari scores correlating with high fever, more vomiting episodes and dehydration.

**Conclusion:**

RV-antigenemia was common in nonbreastfed children with severe RV-diarrhea and correlated with stool RV-load and disease severity.

## Introduction

Rotavirus (RV) is the major worldwide cause of acute gastroenteritis (AGE) with severe dehydrating diarrheain children below 5 years [1]. WHO estimates that RV is responsible for 40% of AGE amongst children (<5 years) and causes 453,000 deaths annually in children under 5 years worldwide, predominately in low-income countries. Currently, two live oral vaccines (RV1; Rotarix and RV5; RotaTeq™) are licensed worldwide [2,3].

The RV belongs to family Reoviridae and generally infectsthevillus enterocytes [4]. However, extraintestinal case reports challenges this concept [5]. Antigenemia, a common phenomenon detected in RV-infected children, is characterized by transient presence of antigen in the blood [6–8] and could explain the mechanism for extraintestinal RV infections. Detection of serum rotavirus antigen ranges from 43–90% in acute phase of the infection. However, the phase of antigenemia is transient as peak levels of RV-antigen are usually seen during early days of infection and are undetectable beyond a week. Simultaneously, RV-RNAemia (presence of RV-RNA in blood) has also been reported earlier [6]. Interestingly, several studies have attempted to find rotavirus RNA/nonstructural proteins from extraintestinal samples [5–7].

The current WHO guidelines recommend continued breastfeeding during diarrhea management [9]. However, protective role of breastfeeding in RV-diarrhea has been questioned [10,11]. Lower immunogenicity and efficacy of the RV vaccines has been demonstrated in lower income countries in Africa and Asia, compared to high income countries. Of interest, studies support that low immunogenicity of RV-vaccines could be due to higher titers plus neutralizing activity of RV-specific-IgA and other nonspecific inhibitorsin breast milk consumed by infants at the time of immunization [11, 12]. Nevertheless, correlation of breastfeeding with systemic manifestations of RV-antigenemia/RV-RNAemia and disease severity in rotavirus-positive patients has not been studied in the Indian population.

## Patients and Methods

### Subjects and sample collection

We have enrolled peri-urban patients <2 years with AGE, attending paediatric facilities either at Nalanda Medical College, Patna or Child Care Center, Patna, Bihar for 2 consecutive years. A total of 173 paired stool and serum samples were collected from immunocompetant, non-RV-vaccinated children; among which 145 were from hospitalised infants with diarrheaand 28 were from age-sex matched healthy infants with no symptoms of diarrheaduring the last 2 weeks before collection (S1 Table). Inclusion criteria for symptomatic cases included passing of ≥3 loose/watery stools within 24 hours and satisfied with one of the following criteria for moderate to severe diarrhea (MSD) viz. a) sunken eyes (confirmed by parent/caretaker); b) skin turgor-loss defined as an abdominal skin pinch with slow or very slow (<2 seconds) recoil; c)intravenous hydration administered/prescribed; d) hospitalization with diarrheaor dysentery. The healthy control group consisted of children who were not suffering from diarrhea before 14 days of case recruitment. Stool/rectal swab samples from all diarrhoeal children were screened for RV. The patients with mixed infections with other enteric viruses/bacteria/parasites were excluded from the study. Two blood samples were retrospectively collected from RV-positive patients: the first within 3 days after admission (acute phase) and the second after 3 weeks of admission (convalescent phase); the serum samples were prepared for further analysis. Extra intestinal presentations were recorded wherever available.

### Ethics statement

The study was performed in accordance with the ethical standards of the Declaration of Helsinki and was approved by the Institutional Review Board, Rajendra Memorial Research Institute of Medical Sciences, Patna. Paired serum and stool specimens were collected after obtaining written informed consent of the parent or guardian prior to enrolling a child. The potential controls were randomly selected from the population and matched to the cases by age, gender, and residence (same/nearby neighbourhood area as the case). A standard questionnaire was used to collect general, demographic, epidemiological and clinical data.

### Assessment of disease severity

Diarrhea was defined as the passage of ≥3 watery stools in a 24-h period [13]. Clinical severity of RV-AGE was assessed by examination of the child and interview of the mother/caregiver by the study paediatrician using the 20 point scale of the Vesikari scoring system [14], based on the frequency and severity of diarrhoea, daily frequency of vomiting, episodes of fever and degree of dehydration. As per the scoring protocol, the episode was considered mild for a score of ≤5, moderately severe for a score of 6–10, and severe for a score of >10.

### Data stratification

We stratified infants, of all groups, by their age. Age stratification was done as follows; Group A: 0–6 months (age of exclusive breastfeeding), Group B: 6–12 months (age of breastfeeding + weaning) and Group C: 13–24 months (age of gradual changeover to adult-like family food). In all groups, infants receiving breast milk during/before their current episode of diarrhea were defined as breastfed and those not receiving anyamount of breast milk represented as non-breastfed infants.

### Detection of RV in stool

Preliminary screening of the collected stool samples for the presence of RV coproantigenwas performed using Rota-Adeno RDT kit as per the manufacturer’s instructions (VIKIA^®^, Rota-Adeno,Biomerieux^®^). Next, for further confirmation, the stool samples were tested for RV by commercial enzyme immunoassay (EIA) Premier^™^RotaClone (Meridian Bioscience Inc., Cincinnati, OH, USA), according to the manufacturer’s instructions. Samples with optical densities >set cut-off point were considered positive while optical densities ≤set cut-off point were taken as negative.

### Viral RNA extraction

From the positive samples, 20% fecal suspensions were used for viral RNA isolation using commercial RNA extraction kit (QIAamp viral RNA MiniKit, Qiagen, Germany) according to manufacturer’s instructions. Complementary DNA (cDNA)was synthesized using random primers (Invitrogen, Life Technologies, USA) and Moloney-Murine-Leukemia-Virus Reverse Transcriptase (M-MLVRT) (Invitrogen, Life Technologies, USA) [15].

### Estimation of viral load in stool

The test cDNA prepared from stool samples were used to estimate viral load in stool by a Realtime RT- PCR method [16]. Briefly, the stool RV-cDNA was used in the VP6-specific real-time PCR using the LightCycler™ (Roche,Germany) with SYBR green-dye and primers [VP6-F(5′GACGGVGCRACTACATGGT3′);VP6-R(5′GTCCAATTCATNCCTGGTGG3′)] resulting in a 379-bp product. RV-load in stool samples was estimated in terms of the PCR cycle or crossing point (C[t] value) and specificity was measured by melting curve analysis.

### Detection of rotavirus antigen and RNAemia in serum specimens

Serum samples retrospectively collected from the RV-positive patients, were screened by EIA for RV-antigenemia and by RT-PCR for RV-RNAemia according to reported methods [6,7,17]. Briefly, undiluted serum specimens (50μL) were tested for RV antigen in serum using Premier^™^RotaClone kit. A modified optical density (OD) cutoffvalue of 0.3 was read at 450 nm wavelength for serum RV-antigen detection[7]. An absorbance OD >cutoff value of 0.3 wasdefined as RV-antigenemia. Levels of RV-antigenemia were normalized for interassay variability against the mean OD value for the negative control serum samples. Simultaneously, RV-RNAs wereextracted from the serum specimens by using a High PureViral Nucleic Acid Kit (Roche Diagnostics, Germany) according to the manufacturer’s protocol. Then, reverse transcription of RV-RNA was performed using Superscript III Reverse Transcriptase(Invitrogen Corporation,USA). The VP7-1’ and Beg9 primers selected for the partial sequence of the VP7gene were studied [18]. The complimentary DNA was amplified with 0.2U Taq DNA polymerase (Fermentas) in 25 μl reaction under the following conditions: initial incubation at 94°C(3min), followed by 35 cycles at 94°C (30sec), annealing at 55°C (30 sec) and extension at 72°C (1min), with a final extension at 72°C (7min). The 395-bp products were checked in2% agarose gel electrophoresis,densitometrically scanned (Quantity-One software; Bio-Rad,USA) and normalized to GAPDH expression.

### Statistical analysis

All statistical analyses were performed using GraphPad PRISM5 Softwareand comparisons were based on the Mann–Whitney U-test or one-way analysis of variance (ANOVA) with a post hoc test. Spearman’s rank correlation and Linear regression were used to assess the correlations. The Pearson correlation was used for data that displayed Gaussian distribution.

## Results

### Prevalence, clinical symptoms and epidemiology in AGE patients

Among 145 faecal samples, 94 (64.8%) were RV-positive and 51 were RV-negative (35.1%). A total of 102 (70.3%) children represented with fever >37°C, 82 (56.5%) with mild/severe dehydration, and 124 (85.5%) with vomiting episodes. Extra intestinal presentations were recorded in 32 diarrheal infants. The Vesikari 20 point scale scoring system for the severity of disease rated the symptomatic children with median value of 10, while healthy children were assigned a score of 0. Diarrhoeal episodes had lasted >4days in 40% of children at the time of admission. Treatment regime with intravenous rehydration followed by oral rehydration was followed for 98 (67.5%) children, whereas 47(32.5%) had received only oral rehydration.

**Fig 1:**
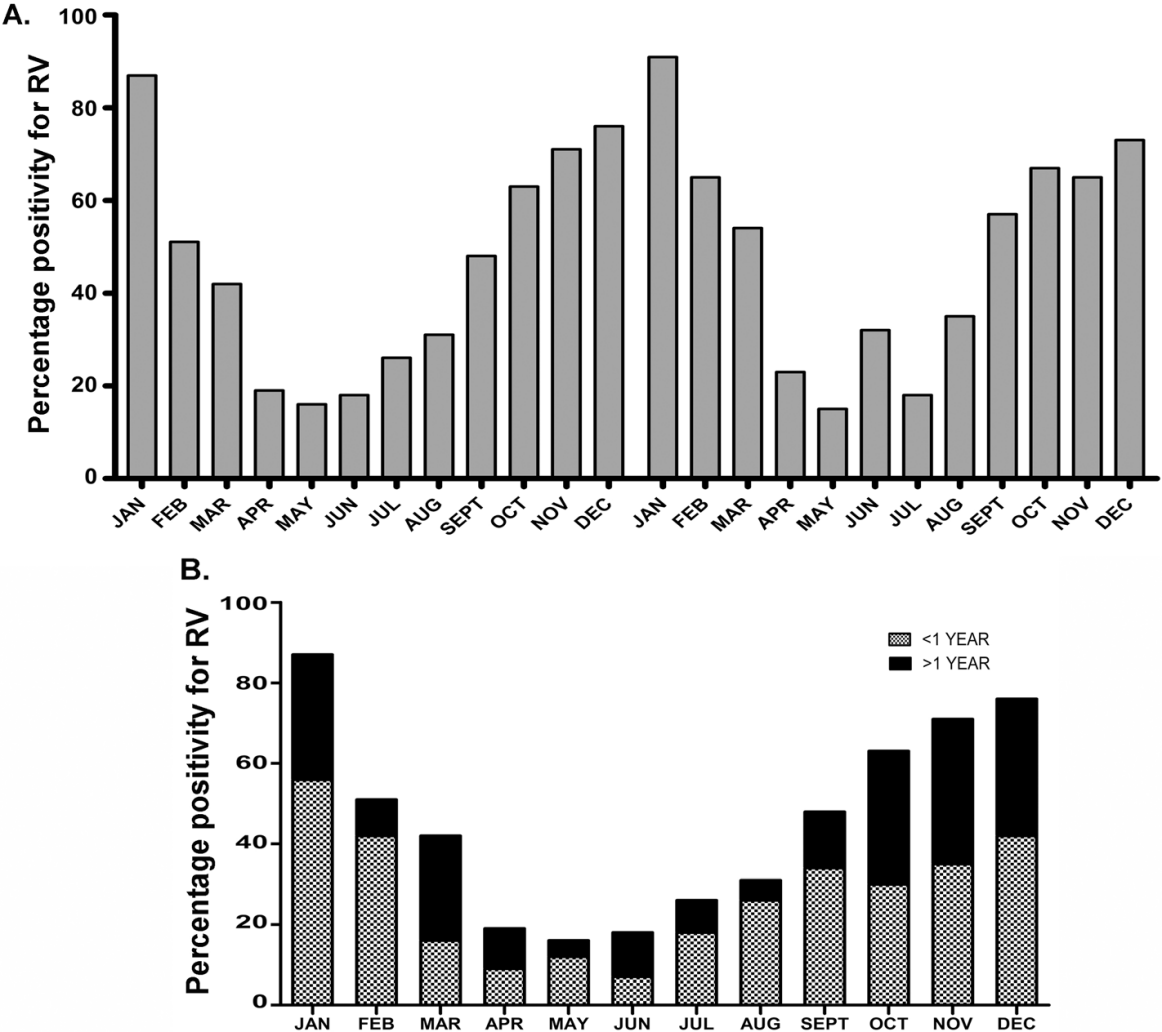
Percentage distribution of Rotavirus-positivity among hospitalalized infants <2 yrs age suffering from severe diarrhea in Patna. (A-B) Temporal distribution of Rotavirus positivity: data of 2 consecutive years (A) and age wise stratified data among all patients (B). (C) Breast-feeding wise distribution of Rotavirus positivity among all patients.

A distinct seasonal variation in RV prevalence was observed, with low levels of positivity (10–20%) throughout the year and surge (75–80%) during the winter months in November to February (Fig 1A). Mostly, children <1 yr age were affected by RV (Fig 1B).

### Breastfeeding Vs Rotavirus antigenemia/RNAemia in children

**Fig 2:**
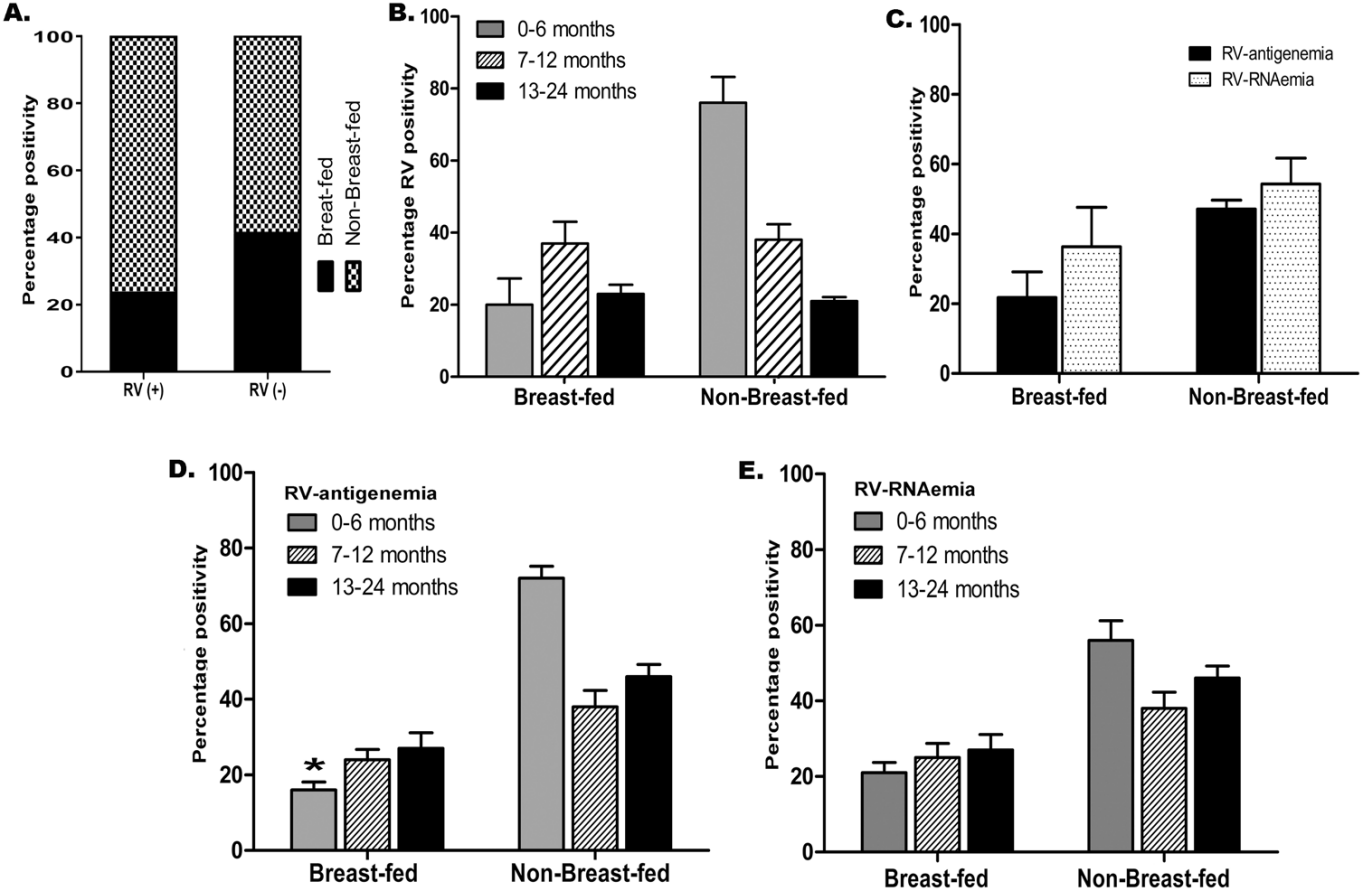
Relation of breast feeding status with Rotavirus infection and related outcome. (A) Breast-feeding wise distribution of Rotavirus positivity among all patients. (B, D-E) Age stratified data among breastfed and non-breastfed infants for Rotavirus positivity (B), Rotavirus antigenemia (D), and Rotavirus RNAemia (E). (C) Percentage positivity for Rotavirus antigenemia and Rotavirus RNAemia among breastfed and non-breastfed infants of the study.

Among the total 145 infants with diarrhea, the prevalence of breastfed infants was 23.4% (34 children) compared to 76.5% (111 children) prevalence of non-breastfed ones (Fig 2A, S1 Table). Notably, RV-diarrhea was most common among the latter group (Fig 2A, S1 Table).

First, the prevalence of RV-diarrhea was observed in the stool samples among the age stratified children groups. In the breast-fed group, RV diarrhea was higher (57%) in the 7–12 months age group compared to the prevalence in other two groups of infants; aged 0–6 months (20%) and 13–24 months (23%) (Fig 2B). Conversely, among the non-breastfed infants, the prevalence of RV-diarrhea was highest (76%) among 0–6 months old group compared to groups of 7–12 months age (18%) and 13–24 months age (6%) (Fig 2B).

Next, the prevalence of RV-diarrhea was observed in the serum samples among infants, with respect to their feeding status. Among the breastfed infants, serum of 21% and 46% presented positivity for RV-antigenemia and RV-RNAemia respectively (Fig 2C). Contrastingly, in non-breastfed, serum of 36% and 54% presented positivity for RV-antigenemia and RV-RNAemia respectively (Fig 2C). It is important to note that RV-antigenemia levels were significantly high among non-breastfed infants (Fig 2D and 2E).

### Rotavirus antigenemiain Indian children

**Fig 3:**
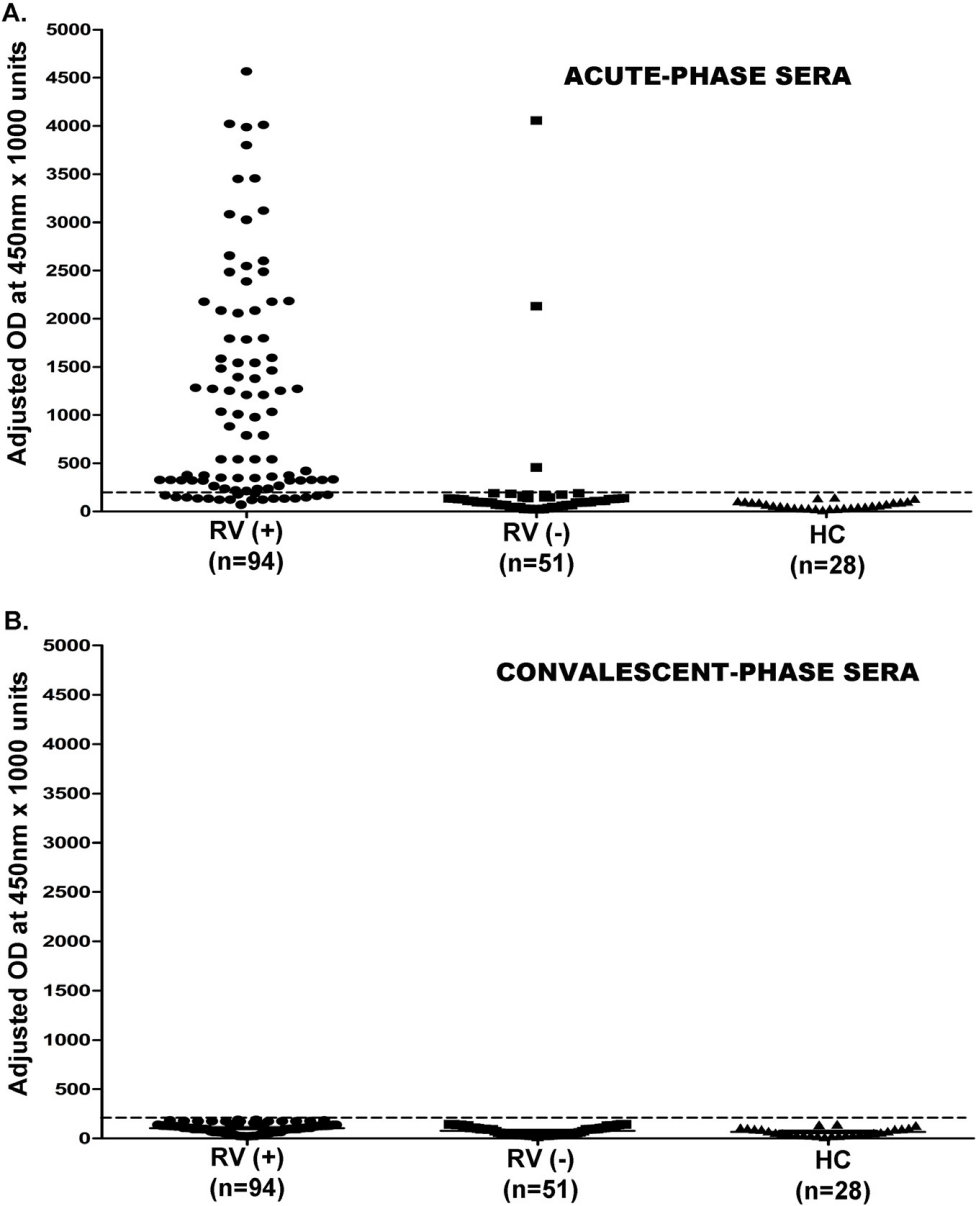
Rotavirus antigenemia levels among the study groups. (A-C) Data demonstrates level of Rotavirus antigenemia in acute-phase (A) and convalescent phase (B) sera in infants suffering from severe diarrhea in comparison with the healthy control group. Paired acute-phase and convalescent phase sera were tested for Rotavirus antigenemia levels, that is denoted by levels of Rotavirus antigen (optical density/O.D.) measured by RV-EIA at 450 nm X 1000 units, as mentioned in the text. Broken lines denote the cut-off value.

The serum VP6 antigen was identified in 67 (71.2%) of 94 RV-positive and in only 3 (5.8%) of 51 RV-negative cases (Fig 3A). Therefore, as reported [19], RV-antigenemia was significantly higher among RV-positive cases (p<0.001). Of note, none of the convalescent phase serum, from 94 RV-positive and 51 RV-negative cases, showed positivity for RV-antigenemia (Fig 3B and 3C).

### Rotavirus RNAemia in Indian children

**Fig 4:**
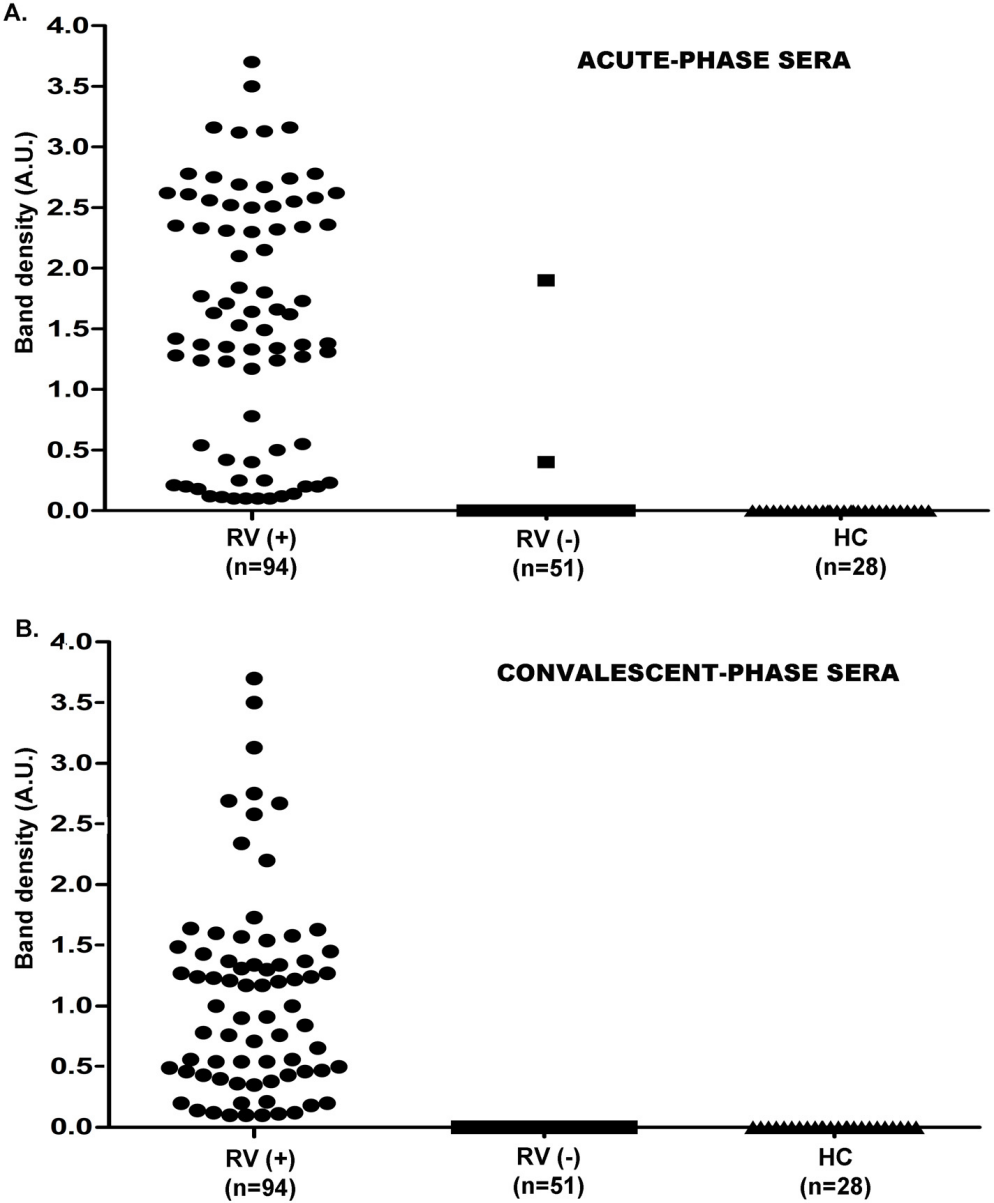
Rotavirus RNAemia levels among the study groups. (A-C) Data demonstrates level of Rotavirus RNAemia in acute-phase (A) and convalescent phase (B) sera in infants suffering from severe diarrhea in comparison with the healthy control group. Paired acute-phase and convalescent phase sera were tested for Rotavirus RNAemia levels, denoted by levels of Rotavirus RNA in form of VP7 gene in reverse-transcriptase PCR calculated by band scanning software, as mentioned in the text.

RV-RNAemia was detected in acute-phase serum of 77 (81.9%) out of 94 RV-positive cases and 2 (3.9%) out of 51 RV-negative cases. (Fig 4A and 4B). Interestingly, some patients with RV-positive diarrhea had confirmatory RT-PCR results for RV-RNAemia,but was negative for RV-antigenemia. RV-antigenemia levels were insignificant in the convalescent phase serum compared to their levels in the acute phase (Fig 3B). However, detectable levels of RV-RNAemia was found in the convalescent phase serum of 79 (84.04%) out of 94 RV-positive cases and 2 (3.9%) out of 51 RV-negative cases (Fig 4B). These two children, with RV-negative stool in EIA, presented positivity for both RV-antigenemia and RV-RNAemia.

### Correlation of Rotavirus antigenemia with stool viral-RNA load and disease severity

**Fig 5:**
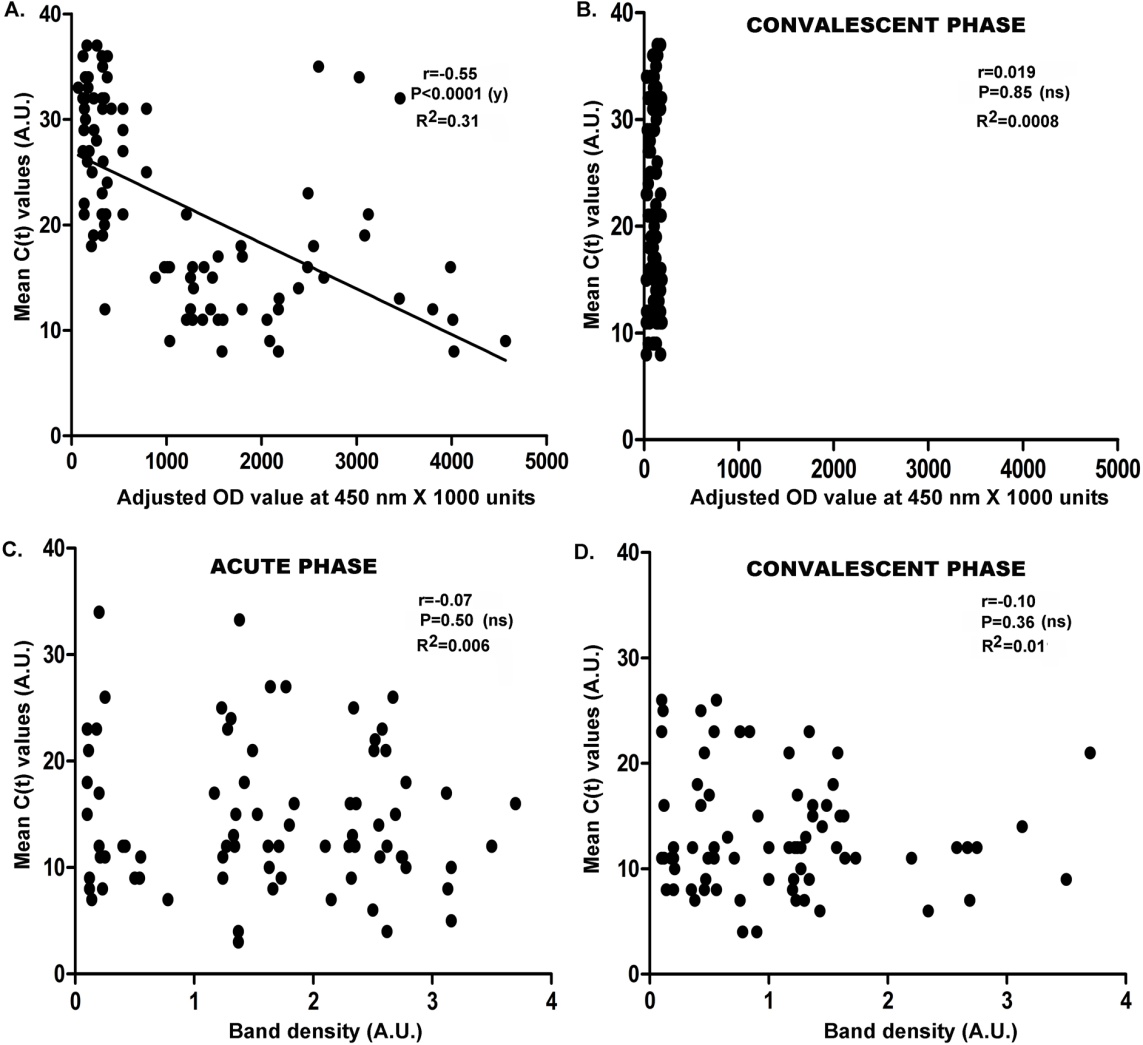
Correlation of Rotavirus antigenemia and RNAemia with stool viral load. (A-D) Correlation of stool viral load with Rotavirus antigenemia (A,B) and Rotavirus RNAemia (C,D) in acute-phase (A,C) and convalescent phase (B,D) serum of infants suffering from acute gastroenteritis and diarrhea. Paired acute-phase and convalescent phase sera were tested for (i) Rotavirus antigenemia levels, that is denoted by levels of Rotavirus antigen (optical density/O.D.) measured by RV-EIA at 450 nm X 1000 units and (ii) Rotavirus RNAemia levels, denoted by levels of Rotavirus RNA in form of VP7 gene in reverse-transcriptase PCR calculated by band scanning software, as mentioned in the text. Stool viral load is represented by C(t) values in real-time RT-PCR.

Next, we compared the RV-antigenemia levels in patient serum (acute and convalescent) with viral load resultsin paired stool samples. The stool viral load result correlated well with levels of RV-antigenemia, i.e. majority of patients with RV-antigenemia results in acute sera(71.2%) had positive RT-PCR results in stool(r = -0.55;CI = -0.68 to -0.39; P<0.0001,R^2^ = 0.31; Fig 5A), but not in convalescent-phase patient sera (r = 0.019;CI = -0.18 to 0.22;P = 0.85,R^2^ = 0.0003,Fig 5B). Thehealthy control individuals were uniformly negative by RT-PCR as well as serum EIA. Interestingly, RV-RNA was not found in stool of 3 children out of 51 RV-negative cases, who were also positive for RV-antigenemia.

**Fig 6:**
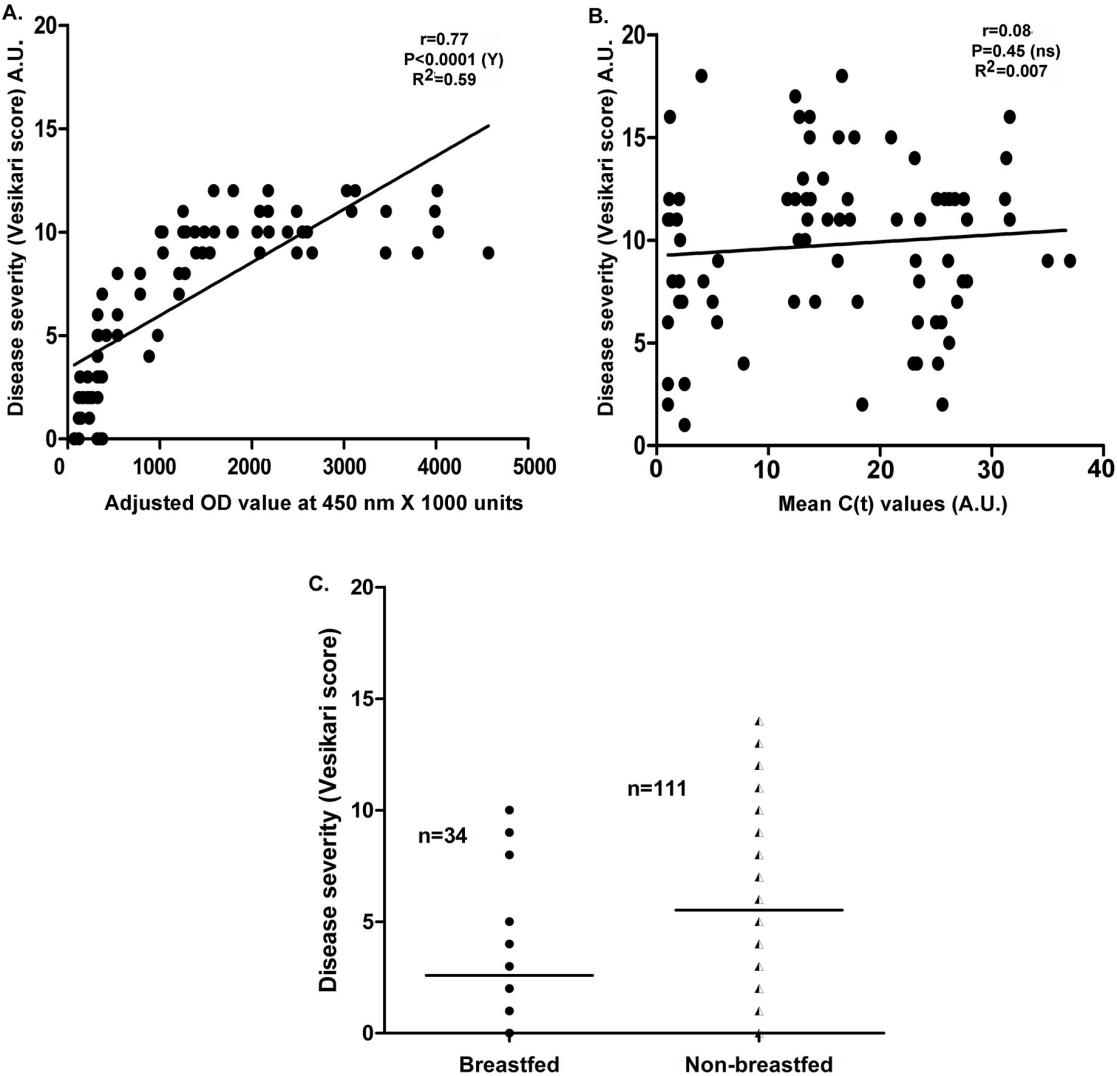
Correlation of Rotavirus antigenemia and RNAemia with disease severity. (A-B) Correlation of disease severity with Rotavirus antigenemia (A) and Rotavirus RNAemia (B) in infants suffering from acute gastroenteritis and diarrhea.(C) Breastfeeding wise stratified data of disease severity among the breastfed and non-breastfed group of infants. Disease severity is represented by Vesikari scores (0–20) according to the 20 point Vesikari scoring system, as mentioned in the text.

Disease severity plus high levels of RV-antigenemiawas recorded in 29 RV-EIA positive infants(30.8%), with Vesikari scores ranging from 9–12 compared whereas low levels of RV-antigenemiashowedVesikari scores ranging from 0–8. Only one patient, reporting intermittent convulsions in the severe group, also presented with high level of RV-antigenemia. Therefore, disease severity was positively correlated with RV-antigenemia (r = 0.74, P<0.0001, R2 = 0.59; Fig 6A). Of interest, all the infants,with high levels of RV-antigenemia and high Vesikari scores, were from the non-breastfed group. Extra-intestinal presentation data was analysed for 32 infants and we found that majority of the patients presented with respiratory tract infections (RTI; 18 with upper RTI and 5 with lower RTI). Only 9 out of 32 patients, with extraintestinal symptoms, also presented with RV-antigenemia.

### Correlation of Rotavirus RNAemia, stool viral RNA load and disease severity

No significant correlation was found for RV-RNAemia positivity in acute (r = -0.07; CI = 0.29 to 0.14, P = 0.5; Fig 5C) or convalescent sera (r = -0.10; CI = -0.32 to 0.12, P = 0.3; Fig 5D) with stool viral-RNA load. Among the 12 infants who were detected positive for RV-RNAemia, five also presented with disease severity, i.e. Vesikari score >10. However, none of the infants,positivefor only RV-RNAemia, presented severity of diarrheal symptoms in terms of Vesikari scores (range: 0–10), in comparison to patients with RV-antigenemia. Therefore, disease severity was not significantly correlated with RV-RNAemia (r = 0.7443, P<0.0001, R2 = 0.5996;Fig 6B).

### Disease severity was related to breast feeding status of the infants

We stratified the data of disease severity according to the breastfeeding status of the infants suffering from AGE with diarrhea. Notably, we found that disease severity was related to breastfeeding habit as the median Vesikari score was high among the non-breastfed group (Fig 6C; S1 Table).

## Discussion

The association between breast feeding and infants health has been noted over past several years. Bioactive components of the human milk either protect from specific pathogens or families of pathogens or confer mucosal immunity to the infant. The present hospital based age-matched case-control study was taken up to analyze the clinical significance of rotavirus antigenemia/RNAemia and disease severity with respect to breastfeeding in Indian infants. Though breastfeeding is recommended duringdiarrhea management, its protective role in rotaviraldiarrhea has been questioned [10.11]. Antigenemia was earlier reported among Indian children [19], but RV-RNAemia/ RV-antigenemiaand disease severity were not evaluated in the context of breastfeeding in the population. In this study, we have enrolled children (<2 yrs age), presenting with AGE and diarrhea, from two tertiary-care hospitals in Bihar, North India (S1 Table). To the best of our knowledge, till date no study has reported AGE with rotavirus infection and its correlation with breastfeeding status of infants in Bihar.

In our study, the rate of RV-antigenemia in the acute-phase serum of RV-positive infants was higher to that of previous studies that ranged from 43%–75% of children with rotavirus AGE [7,19,20]; but was lower than a recent report of 90% [6]. The differences of RV-antigenemia in the reports may be due to the chosen study populations, level of anti-RV IgG, days from onset of diarrhea or the variation between the times of serum collection. Interestingly, it was reported that RV-antigenemia is inversely associated with baseline titres of serum rotaviral-IgG [20]. Additionally, detection of RV-antigenemia also depends on level of viral antigen in the stool [7] and when the blood was drawn [7,17,21,22]. Therefore, the high frequency of RV-antigenemia in our study probably reflects high prevalence of the virus among infants in this zone, high sensitivity of the method used to detect RV-antigenemia in the test samples and/or low transfer of maternal rotaviral serum IgG to the child. Notably, RV-antigenemia was also detected in 5.8% children with RV-EIA negative stool samples. This could imply several possibilities,viz. a) false-negative stool RV-EIA result, b) false-positive RV-antigenemia result and/or c) RV-antigenemiamay not require excretion of the virus/viral antigen in stool. As none of the healthy infants without diarrhea showed positivity for RV-antigenemia, a false-positive result in serum is quite unlikely. It could be postulated that RV-antigenemia does not relate to presence of EIA detectable RV-antigen in the stool.

All RV-EIA negative, but RV-antigenemia positive infants, demonstrated negative results for viral load RT-PCR in stool. Therefore, all of these 3 infants may have past asymptomatic RV infection with undetectable virus or viral antigen in the stool. Interestingly, 28.7% infants were positive for both RV-EIA and RV viral load RT-PCR, but were negative for RV-antigenemia. Therefore, viral antigen or RNA in stool is not always required for RV-antigenemia. Of note, disease severity was associated with RV-antigenemia, as high Vesikari scores was positively related with high levels of RV-antigenemia, but not with RV-RNAemia. Notably, RV-antigenemia level is reported to be directly associated with stool antigen levels but inversely related to specific anti-rotavirus antibodies titres in the serum [17,20].

Immunity induced by natural symptomatic or asymptomatic RV infection can protect from symptomatic RV disease [23]. However, RV-antigenemia and RV-RNAemia indicate induction of both mucosal as well as systemic immune response [5,6,17]. Therefore, considering testing of serum of RV-EIA positive patients for RV-antigenemia may be an additive measure to clinically evaluate the disease. However, our findings indicate that, disease severity and stool viral load is positively related with RV-antigenemia, but not with RV-RNAemia.

In this study, we have attempted to evaluate the correlation of breastfeeding with systemic spread of RV in the form of RV-antigenemia RV-RNAemia in infants with AGE-diarrhea in Bihar, India. The passive protective effect of maternal antibodies, transferred through placenta or through breast milk, may have role against RV infection, at least during early months of breastfeeding [23]. Our results showed that 76.5% of the infants with AGE-diarrhea were from the non-breastfed group and 72% among them presented with RV-antigenemia. Previous report from India demonstrated that, infants whose mothers had high titres of anti-RV-IgA in breast milk remain less affected by RV disease compared to those with low titres of anti-RV-IgA [24]. In a cohort study in Mexico, the serum anti-RV-IgA titre of >1:800, but not high levels of serum anti-RV-IgG, induced 80% protection against the disease [25]. As RV-antigenemia is inversely associated with baseline titres of rotaviral serum IgG in Indian children [20], findings of high rates of RV-antigenemia and RV-RNAemia in nonbreastfed children in the 0–6 months age group may probably reflects low transfer of maternal rotaviral serum IgG to the child through breast milk.

Interestingly, breast milk also contains bioactive components viz. lactoferrin, lactadherin, secretory IgA, lymphocytes, oligosaccharides [26] and human milk glycans [27,28]. Several milk components have crucial nutrients, whose partially digested forms have anti-pathogenic effects and are part of the innate immunity. Two such components are (a) human milk triglycerides and (b) lactoferrins. Interestingly, antimicrobial peptides are abundant in human milk and confer innate protection in the mucosal environment [29, 30]. Lactadherin, a milk-fat globule membrane (MFGM) glycoprotein, has been reported to prevent symptomatic RV infection [29] while the anti-RV antibodies in human milk may also have some role [31]. Exclusive breastfeeding was found to be associated with a lower incidence of RV gastroenteritis [32], non-breastfed infants are therefore vulnerable to RV infections [33]. Interestingly, unlike the human ones, bovine lactadherin is not active against Rotavirus infection [34]. Notably, many human rotaviruses contain a special Asp-Gly-Glu (DGE) motif in VP8 which is required for their adhesion to host integrins establish the infection [33]. Lactadherins competitively bind with the intergrin receptors and prevent RV infection. RV-antigenemia/RV-RNAemia is the systemic spread of the RV leading to severe extra-intestinal complications [5]. Therefore, human breast milk may play a protective role in breastfed infants against spread of RV-antigenemia/RV-RNAemiaandsubsequent disease severity.

Reports also suggest that protection against RV disease can be conferred by neutralizing antibodies in serum [35]. Previous reports have suggested the presence of anti-RV-IgA and neutralizing antibodies in breast milk [36]. We demonstrated that RV-diarrhea was mostly common among the non-breastfed infants, prevalently (76%) in the 0–6 month age group. Studies with oral RV-vaccine indicated that breast-feeding had effects on vaccine efficacy [37]. Although the mechanism of potential suppression of vaccine efficacy by breast milk is debatable;neutralizing antibodies, lactoferrin and lactoadherinmight have a prominent role [38,39]. High Vesikari scores among non breastfed infants (Fig 6C) suggest a protective role of breastfeeding during the early months in infants.

Proper breastfeeding promotion campaigns and counselling programmes are needed to be initiated, especially in low-income countries. Our results are more pertinent from the standpoint of worldwide introduction of live oral rotavirus vaccines and its efficacy in relation to breastfeeding.

## Supporting Information

S1 Table Characteristics of the study subjects with acute gastroenteritis and diarrhea. (DOCX)
