# Supplementary material for: Evaluating the Impact of Breastfeeding on Rotavirus Antigenemia and Disease Severity in Indian Children
Source: PLoS One. 2016 Feb 1;11(2):e0146243. doi: 10.1371/journal.pone.0146243 (PMC4734603; doi:10.1371/journal.pone.0146243)
Supplement: S1 Table — (DOCX) [file pone.0146243.s001.docx]

**S1 Table 1: Characteristics of the study subjects with acute gastroenteritis and diarrhea**

| Characteristics | Breastfed patients  (n= 34) | Non-breastfed patients  (n= 111) |
| --- | --- | --- |
| Mean Age (months) | 7.2 | 4.5 |
| Weight (kgs) | 6.2 | 5.1 |
| Sex (Male/Female) | 13/21 | 38/73 |
| Median duration (days) of illness(IQR) | 3 (2-6) | 6 (3-14) |
| Clinical presentations |  |  |
| Bloody and/or watery stools | 5 (14.7) | 46 (41.4) |
| Recurrent vomiting episodes | 14 (41.7) | 72 (64.8) |
| Abdominal and/or rectal mass | 7 (20.5) | 18 (16.2) |
| Abdominal distensions | 10 (29.4) | 43 (38.7) |
| History of diarrhea (last 14 days) | 11 (32.3) | 49 (44.2) |
| Excessive crying and/or pain | 19 (55.8) | 66 (59.4) |
| Median Vesikari score | 2.7 | 6.4 |
| Rotavirus detected in stool sample | 9 (26.5) | 85 (76.5) |

**NOTE.**

Data are no. (%) of enrolled subjects, unless otherwise indicated.

IQR, interquartile range.
